# E2 allele of the Apolipoprotein E gene polymorphism is predictive for obesity status in Roma minority population of Croatia

**DOI:** 10.1186/1476-511X-10-9

**Published:** 2011-01-18

**Authors:** Hrvojka Marija Zeljko, Tatjana Škarić-Jurić, Nina Smolej Narančić, Željka Tomas, Ana Barešić, Marijana Peričić Salihović, Boris Starčević, Branka Janićijević

**Affiliations:** 1 Department of Internal Medicine, Clinical Hospital «Merkur», Zajčeva 19, 10000 Zagreb, Croatia; 2 Institute for Anthropological Research, Gajeva 32, 10000 Zagreb, Croatia; 3 Clinic Hospital Dubrava, Av. G. Suska 6, 10000 Zagreb, Croatia

## Abstract

**Background and Aims:**

The Roma (Gypsies) are a transnational minority, founder population characterized by unique genetic background modeled by culturally determined endogamy. The present study explores whether the widely found cardiovascular diseases (CVD) risk effects of ACE I/D, APOE (ε2, ε3, ε4), eNOS-VNTR and LEP G2548A polymorphisms can be replicated in this specific population.

**Methods and Results:**

The community-based study was carried on 208 adult Bayash Roma living in rural settlements of eastern and northern Croatia. Risk effect of four CVD candidate polymorphisms are related to the most prominent classical CVD risk phenotypes: obesity indicators (body mass index and waist circumference), hypertension and hyperlipidemia (triglycerides, HDL and LDL cholesterol). For all of them the standard risk cut-offs were applied. The extent to which the phenotypic status is related to genotype was assessed by logistic regression analysis. The strongest associations were found for ε2 allele of the APOE as a predictor of waist circumference (OR 3.301; 95%CI 1.254-8.688; p = 0.016) as well as for BMI (OR 3.547; 95%CI 1.471-8.557; p = 0.005). It is notable that ε3 allele of APOE gene turned out to be a protective genetic factor determining low lipid levels.

**Conclusion:**

The strength of the relation and the similarity of the results obtained for both tested indicators of obesity provide firm evidence that APOE plays an important role in obesity development in the Roma population.

## Findings

The Roma are a transnational minority population of Indian origin fragmented in numerous, often endogamous, smaller groups dispersed throughout Europe, in which the influence of genetic drift is highly emphasized [1-3]. The Roma are also characterized by numerous cultural (life-style) specificities that could contribute to their increased risk for cardiovascular diseases (CVD). The purpose of the present study is, therefore, to examine whether the unique Bayash Roma genetic background exposed some new relations amongst well-known genetic and non-genetic CVD risk factors. The association between classical CVD risk phenotypes (hypertension, obesity and lipid status) and the four widely investigated CVD candidate gene polymorphisms is tested: ACE I/D, APOE (ε2, ε3, ε4), eNOS-VNTR (4,5) and LEP G2548A [4-7].

A total of 230 adult members of the Roma population (78 men and 152 women) aged 20-84 yrs (50.1 ± 14.1) living in rural settlements in Eastern and Northern Croatia were included in the study. All examinees voluntarily participated and before signing the informed consent were informed about the goals, methods and expectations of the study. The study protocol was approved by the local Ethical Committee.

Small samples of peripheral blood (7.5 ml) were collected for genetic and core biochemical analyses (fasting glucose, triglycerides, HDL, LDL and total cholesterol). DNA was extracted from blood samples using the salting out method. Alu I,D (ACE), G-2548A (LEP), VNTR-4,5 (eNOS), and ε2, ε3, ε4 (APOE) polymorphisms were genotyped by applying respective methods [8-10].

Short anthropometry was undertaken [11] and the obesity status was defined using body mass index (BMI = weight (kg)/stature (m)^2^) and waist circumference [12]. Blood pressure measurements were performed in the sitting position, after 10-15 minutes rest, through the use of a mercury sphygmomanometer by an experienced and certified examiner.

Allele frequencies were calculated from genotype frequencies. Deviation from Hardy-Weinberg equilibrium was assessed by exact test. All data (genetic, anthropometric, biochemical) were analyzed univariately using standard statistical methods for quantitative and qualitative variables (data not shown). For each CVD risk phenotype the genotypic odds ratios of selected polymorphisms were estimated using logistic regression analysis including combined effects of sex, age, and other biological covariates. For all studied polymorphisms each homozygote genotype was tested against the other two genotypes. All analyses were performed by SPSS 10.0 statistical package.

The prevalence of CVD risk phenotypes in this sample together with employed standard cut-off values (code 2, for logistic regression) is presented on Table 1. The data demonstrate that the Roma have a serious burden for CVD development that has earlier been connected with a strong cultural basis for obesity, tobacco use and fatty diet [13-15].

**Table 1:** Prevalence of non-genetic cardiovascular disease risk factors in Roma population of Croatia (N = 230).

Variable	Abbreviation	Cut-off value (CODE = 2)	% (for CODE = 2)
Age (years)	AGE50	≥ 50	50
Hypertension (mmHg)	HT	SBP ≥ 140 or DBP ≥ 90 or antihypertensive therapy	44
Glucose (mmol/L)	FG	≥ 6	12
Triglycerides (mmol/L)	Triglyc_C	≥ 1.7	52
Total cholesterol (mmol/L)	Cholest_C	≥ 5.0	78
LDL cholesterol (mmol/L)	LDL_C	≥ 3.0	82
HDL cholesterol (mmol/L)	HDL_C	≤ 1.0 (males); ≤ 1.2 (females)	16
Body mass index (kg/m^2^)	BMI25	BMI ≥ 25	58
Waist circumference (cm)	Waist_C	Waist ≥ 80 (females); ≥ 94 (males)	63

Genotype and allele frequencies (N & %) for all four examined CVD candidate polymorphisms (ACE I/D, eNOS-VNTR, LEP G2548A and APOE (ε2, ε3, ε4)) in Croatian Roma are presented in Table 2. Since no departure from HW expectations existed we assumed homogeneity of the studied population with respect to all four studied polymorphisms.

**Table 2:** Genotype and allele frequencies of ACE I/D, eNOS-VNTR, LEP_G2548A, and APOE polymorphisms in Croatian Roma

Polymorphism	N (%)					HWE p
*ACE ID*						
Genotypes	*II*	*ID*	*DD*			
	47 (20.7)	112 (49.3)	68 (30.0)			0.997
Alleles	*I*	*D*				
	206 (45.4)	248 (54.6)				

*eNOS VNTR*						
Genotypes	*44*	*45*	*55*			
	5 (2.4)	42 (20.1)	162 (77.5)			0.328
Alleles	*4*	*5*				
	52 (12.4)	366 (87.6)				

*LEP G2548A*						
Genotypes	*GG*	*AG*	*AA*			
	25 (11.6)	96 (44.7)	94 (43.7)			0.998
Alleles	*G*	*A*				
	146 (34.0)	284 (66.0)				

*APOE*						
Genotypes	E2E3	E2E4	E3E3	E3E4	E4E4	
	30 (14.4)	12 (5.8)	109 (52.4)	52 (25.0)	5 (2.4)	0.189
Alleles	E2	E3	E4			
	42 (10.1)	300 (72.1)	74 (17.8)			

The relations of all CVD phenotypes with four candidate polymorphisms were tested by logistic regression analysis. The independent variables were tested as quantitative and qualitative ones and the better variant for each phenotype was used. The best models of logistic regression analyses are presented in Table 3.

**Table 3:** Logistic regression models for waist circumference (Waist_C), body mass index (BMI25), fasting triglyceride (Triglyc_C) and LDL cholesterol (LDL_C) concentration in blood

Tested variable	Predictor variables	OR (95% CI)	p
**Waist circumference (Waist_C)**:	Sex (males are referent)	3.122 (1.475 - 6.607)	**0.003**
1 = < 80 cm (F), < 94 cm (M);	Age (younger than 50 yrs are referent)	0.728 (0.349 - 1.517)	0.397
2 = ≥ 80 cm (F), ≥ 94 cm (M)	E2 allele of APOE gene (persons who do not have the genotypes: E2E3 or E2E4 are referent)	3.301 (1.254 - 8.688)	**0.016**
-2 Log Likelihood = 198.021; Cox & Snell R^2 ^= 0.251; Nagelkerke R^2 ^= 0.344	Glucose in blood (mmol/L)	1.959 (1.234 - 3.112)	**0.004**
	Triglyceride in blood (mmol/L)	1.359 (1.043 - 1.770)	**0.023**
	LDL cholesterol in blood (mmol/L)	1.583 (1.043 - 2.403)	**0.031**
	HDL_C (>1.0 (M) and >1.2 (F) are referent)	5.505 (1.542 - 19.650)	**0.009**
**Body mass index (BMI25)**:	Sex (males are referent)	1.027 (0.506 - 2.086)	0.940
1 = BMI < 25;	Age (years)	0.979 (0.955 - 1.003)	0.080
2 = BMI ≥ 25	E2 allele of APOE gene (persons who do not have the genotypes: E2E3 or E2E4 are referent)	3.547 (1.471 - 8.557)	**0.005**
-2 Log Likelihood = 231.095; Cox & Snell R^2 ^= 0.181; Nagelkerke R^2 ^= 0.242	Glucose in blood (mmol/L)	1.323 (0.964 - 1.816)	0.083
	Triglyceride in blood (mmol/L)	1.309 (1.041 - 1.646)	**0.021**
	LDL cholesterol in blood (mmol/L)	2.031 (1.369 - 3.014)	**0.000**
	HDL_C (>1.0 (M) and >1.2 (F) are referent)	4.041 (1.521 - 10.737)	**0.005**

**Triglyceride concentration (Triglyc_C)**:	Sex (males are referent)	1.345 (0.702 - 2.578)	0.372
1 = triglyceride concentration < 1.7 mmol/L;	Age (younger than 50 yrs are referent)	1.678 (0.911 - 3.091)	0.097
2 = triglyceride concentration ≥ 1.7 mmol/L	E3_55 (persons who do not have the combination:		
-2 Log Likelihood = 241.753; Cox & Snell R^2 ^= 0.095; Nagelkerke R^2 ^= 0.127	E3 (APOE) + 55 (eNOS) are referent)	0.396 (0.199 - 0.785)	**0.008**
	BMI (kg/m^2^)	1.046 (0.992 - 1.103)	0.094
	Index (= LDH/HDL)	1.337 (0.922 - 1.938)	0.125

**LDL cholesterol concentration (LDL_C)**:	Sex (males are referent)	2.682 (1.155 - 6.226)	**0.022**
1 = LDL concentration < 3.0 mmol/L;	Age (younger than 50 yrs are referent)	2.428 (1.024 - 5.760)	**0.044**
2 = LDL concentration ≥ 3.0 mmol/L	E3_55_II (persons who do not have the combination:		
-2 Log Likelihood = 144.692; Cox & Snell R^2 ^= 0.132; Nagelkerke R^2 ^= 0.220		0.302 (0.112 - 0.815)	**0.018**
	E3 (APOE) + 55 (eNOS) + II (ACE I/D) are referent) BMI (kg/m^2^)	1.153 (1.058 - 1.256)	**0.001**

The most significant relation among the investigated CVD risk phenotypes and the four candidate polymorphisms was found between APOE ε2 and obesity indicators (variables: Waist_C and BMI25). The best logistic model explains 34.4% of Waist_C variance and shows that ε2 allele (i.e. genotypes ε2ε3 and ε2ε4) increases the risk of high Waist_C phenotype by 3.301 times (95% CI 1.254 - 8.688; p = 0.016). Similarly significant findings have been obtained for the variable BMI25 where the best model shows that ε2 allele increases the risk of having BMI >25 by 3.547 times (95% CI 1.471-8.557; p = 0.005).

Neither allele, genotype nor their combination presented itself as a risk for hypertension, elevated triglycerides, total, LDL or HDL cholesterol. However, two significant findings emerged and they were related to decreased risk of hyperlipidemia. This protective relation showed the combination of allele ε3 (APOE) and genotype 55 (eNOS) for the triglyceride level and the same combination with addition of genotype II (ACE) for LDL. 141 participants (71.6%) of the study had the former protective combination and 31 participants (15.8%) had the later combination.

Numerous studies revealed the influence of APOE genotypes on lipid and lipoprotein levels. Generally it was shown that ε2 allele is associated with decreased and ε4 with increased total cholesterol level [16-18]. The results of the meta-analysis [17] also indicated a consistent relationship between plasma triglyceride levels and apoE phenotype in different populations: triglyceride concentrations were significantly higher in ε2ε2, ε2ε3, ε3ε4 and ε2ε4 than in ε3ε3 subsets which are consistent with the results of the present study.

Some studies showed that ε2 allele carriers have decreased apoE receptor binding capacity in liver, and transport of VLDL and chylomicrons from blood to hepatocytes, than ε3 and ε4 carriers [16,19]. The association between high saturated fat intake with increased VLDL cholesterol, decreased HDL cholesterol and smaller LDL sizes in ApoE ε2 carriers [20] suggests that in ε2 carriers a high-saturated fat intake may result in increased VLDL production and delayed clearance. It seems that modulation of dietary cholesterol absorption, dietary cholesterol, obesity, sex and even hypolipidemic drug treatment may also be influenced by APOE polymorphism [21].

Therefore, it is possible that gene-nutrition interactions [22,23] are responsible for the observed association between ε2 allele and obesity. Namely, our preliminary study has shown relatively high prevalence of overweight individuals (58%) among the Croatian Roma which was connected with unhealthy eating habits resulting from poverty. They included irregular meals, high consumption of animal fat, coffee and alcohol and low consumption of meat and milk products, fresh vegetables and fruit [13] which is comparable to the other Roma groups in Central European countries [24,25]. Although the most prominent CVD risk factor in Croatian Roma population is related to the extremely high rates of smoking habit that is present in 72% men and 69% women [14], it should be stressed out that they are also characterized by a high prevalence of increased levels of LDL cholesterol exceeding the recommended levels in as much as 82% of the Roma sample. We hypothesize that the nutritional habits provoke a metabolic response [26,27] that could reveal a relative susceptibility in ε2 carriers.

The study's weakness is its limited sample size and it must therefore be regarded as preliminary to larger investigations. These will include the analysis of family data directed towards confirming linkage between APOE ε2, ε3, ε4 polymorphism and obesity phenotype. Additionally, the apolipoprotein E gene-nutrition interactions are planned to be examined in more detail.

The main strength of the study is that it was performed without presumptive hypothesis about the effect of particular genotypes and thus the possible impact of each genotype was explored alone and in combination with other genetic and environmental factors. This approach enabled the effect of E2 allele to emerge.

In conclusion, APOE polymorphism is a major determinant of CVD risk in the Croatian Roma acting on several phenotypic risk traits - obesity indicators and lipid levels (LDL and triglycerides). While the protective role of ε3 allele and the risk of ε2 and ε4 is not a novel finding, the strength of the relation of ε2 allele with obesity indicators is a remarkable result of the present study.

## Competing interests

The authors declare that they have no competing interests.

## Authors' contributions

HMZ participated in the design of the study and drafted the manuscript. TŠJ, BS and NSN participated in the design of the study, contributed to acquisition of data and its interpretation, performed the statistical analysis and helped to draft the manuscript. ŽT, AB, and MPS carried out the molecular genetic study, participated in the sequence alignment and contributed to acquisition of data and its interpretation. BJ conceived the study, participated in its design and coordination and contributed to acquisition of data and its interpretation. All authors read and approved the final version of the manuscript.
